# RCPedia: a global resource for studying and exploring retrocopies in diverse species

**DOI:** 10.1093/bioinformatics/btae530

**Published:** 2024-09-06

**Authors:** Helena B Conceição, Rafael L V Mercuri, Matheus P M de Castro, Daniel T Ohara, Gabriela D A Guardia, Pedro A F Galante

**Affiliations:** Hospital Sirio-Libanes, São Paulo 01308-060, Brazil; Interunidades em Bioinformática, Universidade de São Paulo, São Paulo 05508-000, Brazil; Hospital Sirio-Libanes, São Paulo 01308-060, Brazil; Interunidades em Bioinformática, Universidade de São Paulo, São Paulo 05508-000, Brazil; Hospital Sirio-Libanes, São Paulo 01308-060, Brazil; Department of Biochemistry, University of São Paulo, São Paulo, 05508-000, Brazil; Hospital Sirio-Libanes, São Paulo 01308-060, Brazil; Hospital Sirio-Libanes, São Paulo 01308-060, Brazil; Hospital Sirio-Libanes, São Paulo 01308-060, Brazil

## Abstract

**Motivation:**

Gene retrocopies arise from the reverse transcription and genomic insertion of processed mRNA transcripts. These elements have significantly contributed to genetic diversity and novelties throughout the evolution of many species. However, the study of retrocopies has been challenging, owing to the absence of comprehensive, complete, and user-friendly databases for diverse species.

**Results:**

Here, we introduce an improved version of RCPedia, an integrative database meticulously designed for the study of retrocopies. RCPedia offers an extensive catalog of retrocopies identified across 44 species, which includes 13 primates, 4 rodents, 6 chiropterans, 12 other mammals, 4 birds, turtles, lizards, frogs, zebrafish, and *Drosophila*. The database offers the most complete compilation of retrocopies per species, accompanied by detailed genomic annotations, expression data, and links to other data portals. Furthermore, RCPedia features a streamlined representation of data and an efficient querying system, establishing it as an invaluable tool for researchers in the fields of genomics, evolutionary biology, and transposable elements (TEs). In summary, RCPedia aims to enhance the investigation of retrocopies and their pivotal roles in shaping the genomic landscapes of diverse species.

**Availability and implementation:**

RCPedia is available at https://www.rcpediadb.org.

## 1 Introduction

Gene retrocopies (retrocopies) are gene copies formed through the reverse transcription of mRNAs, followed by their genomic integration (Kaessmann *et al.* 2009). The formation of retrocopies involves several molecular steps, primarily driven by enzymes encoded by long interspersed nuclear element-1 (LINE-1 or L1). Due to the absence of the parental gene’s regulatory elements required for transcription and the presence of mutations in many copies, retrocopies are often classified as processed pseudogenes. However, with the recent advancements in enabling technologies, an increasing number of retrocopies have been recognized as functional (Navarro and Galante 2015; Casola and Betrán 2017; Cheetham *et al.* 2020). Their significance manifests in several aspects: (i) gene regulation, influencing the transcription of nearby or parental genes (Poliseno *et al.* 2010); (ii) genetic innovation, with some retrocopies transcribed and translated into functional retrogenes distinct from parent genes (Burki and Kaessmann 2004); (iii) contribution to genomic diversity, particularly through polymorphisms in populations (Schrider *et al.* 2013); and (iv) disease associations, including cancer, through gene function disruption or misregulation (Karreth *et al.* 2015; Bim *et al.* 2019). Thus, studying retrocopies is crucial for understanding the complex aspects of species evolution and innovation.

The advancement of genomics and related fields has been significantly accelerated by web tools and databases. These resources are indispensable for managing, analyzing, and interpreting extensive genomic datasets, facilitating groundbreaking discoveries. However, there is a noticeable scarcity of databases dedicated to retrocopies. Currently, only two such dedicated and active databases exist: RCPedia (Navarro and Galante 2013) and RetrogeneDB (Kabza *et al.* 2014). RCPedia initially focused on human and primate retrocopies but was limited in species coverage. RetrogeneDB, while valuable, employs a stringent identification pipeline resulting in a limited number of retrocopies per species. Additionally, expression data are restricted to few tissues, even in well-studied species like humans.

This improved version of RCPedia (https://www.rcpediadb.org) represents a significant advancement over its predecessor. Building on the original platform’s success, the new RCPedia version was built upon improved computational pipelines, with a more sensitive aligner and additional filters (for details, see Section 3), along with up-to-date genomic references and gene annotations, offering an expanded and comprehensive catalog of retrocopies across 44 species, along with extensive RNA-Seq expression data. All features are available through a user-friendly web interface, enhancing accessibility and utility for the research community. This expansion and refinement of RCPedia are poised to substantially impact the study of retrocopies and their role in shaping the genomic landscape across diverse species.

## 2 Data retrieval and curation

### 2.1 Data sources

**Figure 1.**
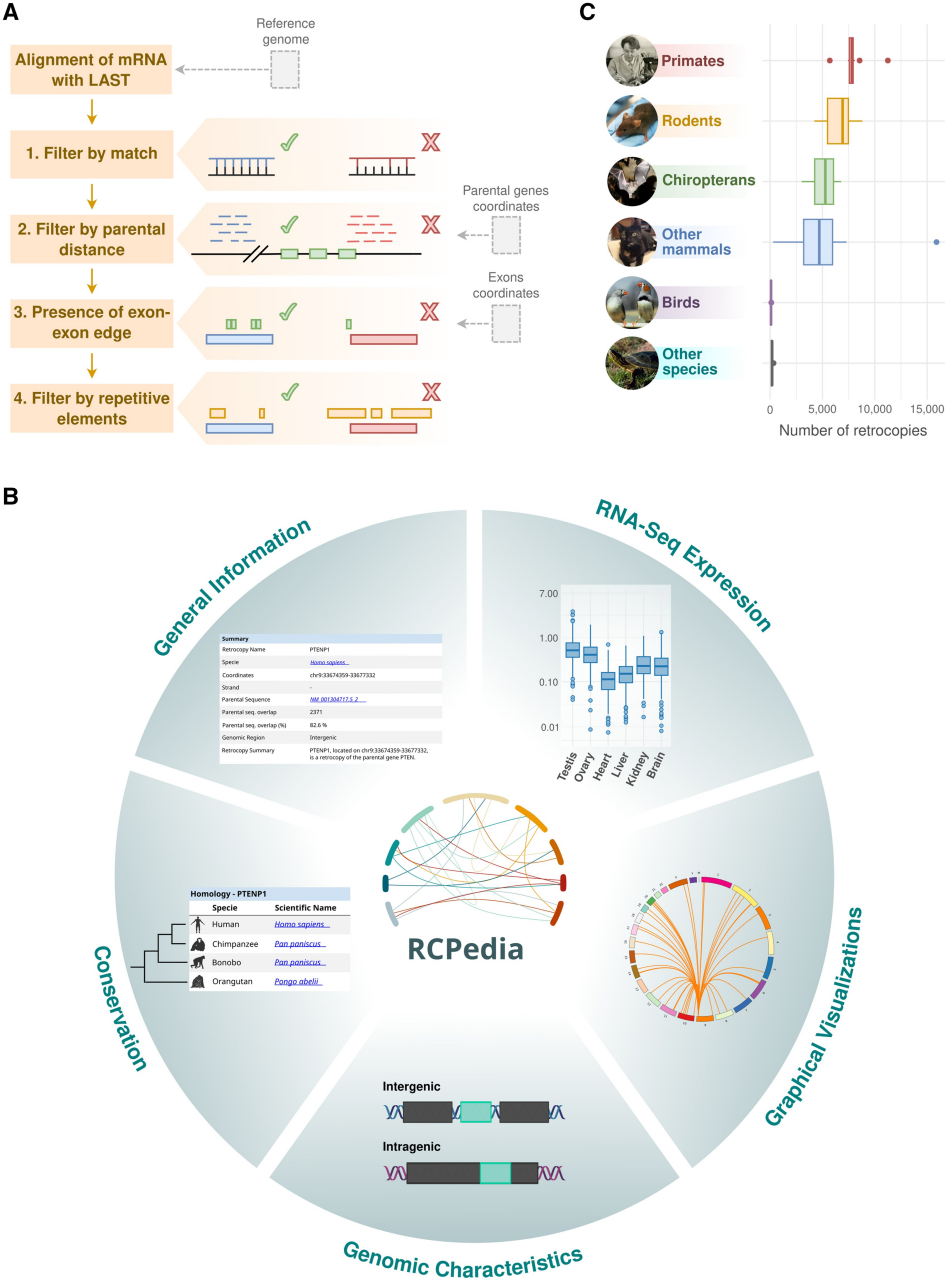
Overview of RCPedia’s features and data. (**A**) The retrocopy identification workflow in RCPedia. This schematic delineates the algorithm’s main steps for identifying retrocopies, beginning with mRNA alignment and followed by sequential filtering based on a match threshold, parental gene distance, presence of exon-exon junctions, and exclusion of repetitive elements. (**B**) The user interface and data presentation in RCPedia. Illustrated here is a sample of the comprehensive information available for each retrocopy and its parental gene, including summary statistics, genomic coordinates, cross-species homology, RNA-seq expression levels, and genomic context. (**C**) Distribution of retrocopy numbers across different species groups. The box plots demonstrate the range and median values of retrocopies identified within groups of primates, rodents, chiropterans, other mammals, birds, and additional vertebrate species.

RCPedia includes data from 44 species, from humans to *Drosophila* (Supplementary Table 1). As with the previous version, the identification of retrocopies for each species is grounded in two key datasets: A well-assembled reference genome sequence and a set of known annotated genes. Of these, 34 genomes are based on long-read assemblies (Supplementary Table 2), and 38 are assembled at the chromosome level. The remaining six genomes (bats) are assembled at the scaffold level, but their quality is comparable to chromosomal assemblies (Jebb *et al.* 2020). Given the reliable assembly quality of all genomes used, the number of retrocopies identified in the species present in RCPedia is not influenced by these features (Supplementary Fig. 1). Figure 1A presents a schematic overview of the retrocopy identification process. A comprehensive description of our algorithm for retrocopy identification is provided in the Supplementary Material (and section below). The versions of the genomes and corresponding reference transcriptomes utilized are detailed in Supplementary Table 2. Retrocopy expression data are derived from publicly available RNA-seq datasets; the complete list of datasets used can be found in Supplementary Table 3.

### 2.2 Identifying expressed and orthologs retrocopies

A crucial step for retrocopies to become functional is the acquisition of expression (Navarro and Galante 2015; Carelli *et al.* 2016). To provide the most comprehensive and accurate data on retrocopy expression, across 37 of the 44 species, we reprocessed RNA-Seq data from 878 samples. For humans and mice, we utilized precalculated expression data from two major databases: GTEx and ARCHS4. To retrieve the precalculated expression data for humans and mice, we mapped the genomic coordinates of the identified retrocopies to the genomic coordinates of genes annotated in Ensembl, whenever available. In humans and mice, we observed retrocopy expression in 154 202 samples spanning 35 tissues. For the other species, we obtained raw RNA sequencing data from paired-end experiments performed on Illumina platforms with an average read length of at least 100 base pairs from the GEO database (https://www.ncbi.nlm.nih.gov/geo/). Raw files were preprocessed using fastp (Chen *et al.* 2018) (default parameters), and expression quantification was performed using Kallisto (Bray *et al.* 2016) (default parameters with bootstrap = 100) with the reference transcriptomes constructed using both RefSeq-annotated sequences and the sequences from the identified retrocopies. Expression was noted in six preselected tissues (brain, heart, liver, kidney, ovary, and testis). These tissues were specifically chosen due to their biological significance, the abundance of available RNA-seq data across many species, and their tendency to express retrocopies.

## 3 Database implementation

Similar to its previous version (Navarro and Galante 2013), the RCPedia database is built on a relational database structure using MariaDB (https://mariadb.org/). The website was developed primarily in PHP (http://www.php.net), utilizing CakePHP (http://cakephp.org) as the framework for an efficient Model-View-Controller front-end. Genomes and gene annotations were processed using a combination of Perl (http://www.perl.org), Python, and in-house developed shell script algorithms.

For retrocopy identification, we refined our previously developed methods (Navarro and Galante 2013, 2015). Briefly, coding transcripts from RefSeq were aligned against their respective reference genomes using LAST (Kiełbasa *et al.* 2011) with specified parameters. Unlike BLAT (Kent 2002), which was used previously and employs fixed-length seeds, LAST uses adaptive seeds that dynamically adjust their length based on the characteristics of the input sequences (Kiełbasa *et al.* 2011). This feature enhances LAST’s sensitivity, specificity, speed, and efficiency, making it ideal for genome mapping of mRNA sequences that contain insertions, deletions, and substitutions, a crucial step in aligning parental sequences to the retrocopy region. Additionally, we now employ a well-structured four-step strategy for identifying retrocopies, Fig. 1A: (i) selecting alignments with at least 120 matched nucleotides, which is half the average size of human exons based on GENCODE (version 40); (ii) excluding alignments of messenger RNA in the original protein-coding gene loci or up to 200 000 bp, a strategy to avoid false positives arising from tandem duplications (Bailey *et al.* 2001); (iii) mapping exon-exon boundary positions from parental genes onto the alignments, retaining only those depicting an intronless region nearest the 3′ end of the messenger RNA; and (iv) removing alignments that are composed by more than 40% of transposable elements (TEs) like LINEs and SINEs. The final retrocopy set was defined by selecting all remaining alignments and grouping those mapped to the same genomic locus (detailed in Supplementary Material).

## 4 Database query interface and output visualization

### 4.1 The query system

RCPedia features both a direct and an advanced query system, designed for ease of use and speed (Supplementary Fig. 2). Users can efficiently perform searches using commonly utilized identifiers. Queries can be made using the name of the parent gene [e.g. glyceraldehyde-3-phosphate dehydrogenase (GAPDH)], the retrocopy name (e.g. PTENP1), genomic positions (e.g. chr17:17000000–18000000), or across an entire chromosome (e.g. chr17). Additionally, the system supports queries based on other gene identifiers, such as ENSEMBL gene ID (e.g. ENSG00000232230), and transcript identifiers, including Refseq IDs (e.g. NM_001281497.2), Supplementary Fig. 3.

### 4.2 Results

RCPedia effectively presents information on retrocopies and parental genes for all 44 species analyzed. The database includes detailed annotation information for both genes and retrocopies, based on NCBI gene data. For humans, when a retrocopy is already named, we adhere to this annotation. Unannotated retrocopies are named following the pattern “parental gene name” + P [number] (e.g. FTLP17). Additionally, we provide their genomic locations, sequences, and graphical visualizations through Circos plots. RCPedia also includes data on expression [measured in transcripts per million (TPM) and the logarithm of this value] and the conservation of each retrocopy across species. This information is graphically represented in Fig. 1B and Supplementary Fig. 4. Figure 1C summarizes the number of retrocopies identified in each species group. Overall, Fig. 1C shows that the number of retrocopies varies considerably across the groups. Primates and rodents exhibit higher and also variable numbers of retrocopies, while birds and other vertebrates (species) show fewer retrocopies with less variability. As pointed out in the literature (Navarro and Galante 2015) and confirmed in our data (Supplementary Fig. 5), a higher number of retrocopies is associated with a higher activity of LINE1 elements. Moreover, the presence of outliers in primates and rodents may suggest that certain species within these groups may possess unique evolutionary pressures or experience additional mechanisms influencing retrocopy numbers. Interestingly, birds and platypuses (monotremes), species with a high presence of LINE-2—elements whose replication machineries are not expected to retroduplicate other mRNAs in trans (Wei *et al.* 2001)—have a low number of retrocopies. Users can easily search for retrocopy numbers per species using RCPedia, as detailed in Supplementary Fig. 6.

We compared the current version of RCPedia (also referred to as RCPedia 2.0) with other retrocopy/pseudogene databases to highlight its unique advantages. In a coordinate-based comparison of retrocopies in the human genome with RCPedia 1.0 (previous version), RetrogeneDB, Gencode V40, and UCSC Track RetroGenes V9, we found that 7966 (98.6%) of retrocopies from RCPedia 2.0 were identified in at least one other annotation, 7640 (94.6%) in two or more, 6645 (82.2%) in three or more, and 3568 (44.2%) by all methodologies (Supplementary Fig. 7). Comparisons with Gencode V40 and UCSC Track RetroGenes V9 revealed significant methodological differences. RetroGenes V9’s less stringent filtering results in a putative high false positive rate (5789 specific retrocopies, Supplementary Fig. 7), while Gencode V40’s combination of computational and manual annotations provides high accuracy but is challenging to scale (Supplementary Fig. 7).

In comparing RCPedia with RetrogeneDB, we found 4254 shared retrocopies, representing 93.2% of RetrogeneDB’s retrocopies (Supplementary Fig. 8). RCPedia identifies 3712 retrocopies not found by RetrogeneDB, but also found by other annotations (Supplementary Fig. 7), representing an 81.3% increase. RetrogeneDB’s stringent criteria exclude many true retrocopies by requiring at least two exon-exon junctions (Supplementary Fig. 8). Compared to RCPedia 1.0, there was an 85.4% overlap with RCPedia. Methodological differences, such as the transition from the BLAT aligner and hg19 assembly in RCPedia 1.0 to stricter filters in current RCPedia, explain the incomplete overlap. Despite these changes, 90.33% of additional retrocopies identified in the current version of RCPedia were annotated by at least one other method (Supplementary Figs 7 and 8).

The high overlap with other databases and the consensus identification of around 8000 retrocopies in the human genome indicate RCPedia’s reliability and comprehensiveness, making it a valuable resource for researchers.

## 5 Using RCPedia

To demonstrate the utility of RCPedia, we focused on the retrocopies of the GAPDH gene. GAPDH, a gene encoding a crucial enzyme in cellular metabolism, is involved in various cellular processes and is commonly used as a housekeeping gene due to its stable expression across tissues. However, it is also one of the most frequently retrocopied genes in mammals, a significant aspect often overlooked. RCPedia reveals 1659 retrocopies of GAPDH across 29 species. Notably, rats, mice, and humans have 261, 100, and 53 GAPDH retrocopies, respectively, contributing to a total of 415 copies. Intriguingly, 789 of these retrocopies are conserved across other animals in our dataset, and 100% of species exhibit expression (> 0.1 TPM) in at least one of their GAPDH retrocopies in one or more samples. Specifically, in humans and mice, 52 and 96 retrocopies, respectively, are expressed (Supplementary Fig. 9).

## 6 Future perspectives

In future updates of RCPedia, we aim to enrich the database with data that strengthen the evidence for retrocopy expression, shedding light on the regulatory mechanisms and further functional roles of these gene duplicates. By leveraging datasets from large-scale public initiatives, such as ENCODE, we aim to augment our database with cap analysis of gene expression (CAGE) for pinpointing transcription start sites of retrocopies in organisms like humans and mice. Additionally, we plan to integrate an assay for transposase-accessible chromatin sequencing (ATAC-seq) to assess chromatin accessibility at retrocopy insertion sites, along with chromatin immunoprecipitation sequencing (ChIP-Seq) to examine markers of transcriptional activity, including RNA polymerase II occupancy and histone modifications associated with retrocopy expression. As the sequencing and annotation of genomes from more species improve, our efforts will continue in the identification and integration of retrocopies into RCPedia. Particularly for humans, we intend to expand our database to include RNA-Seq expression data from various pathological contexts, including cancer, to provide a more comprehensive resource for understanding retrocopy dynamics in disease.

## 7 Conclusion

RCPedia emerges as a well-organized, user-friendly resource featuring a streamlined graphical interface, focusing on the study of retrocopies in 44 species. This database significantly complements existing resources such as RetrogeneDB, Pseudogene.org, and retroFinder. It fills critical gaps by providing a dedicated platform for comprehensive retrocopy identification and enhances data integration, browsing, and visualization capabilities. We are confident that this updated and enhanced version of RCPedia will greatly facilitate more extensive and in-depth investigations of retrocopies across diverse species. This advancement is expected to deepen our understanding of retrocopies’ roles in genomic evolution, gene expression regulation, and their association with diseases.

## Supplementary Material

btae530_Supplementary_Data
