# Supplementary material for: RCPedia: a global resource for studying and exploring retrocopies in diverse species
Source: Bioinformatics. 2024 Sep 6;40(9):btae530. doi: 10.1093/bioinformatics/btae530 (PMC11387616; doi:10.1093/bioinformatics/btae530)
Supplement: btae530_Supplementary_Data [file btae530_supplementary_data.zip › SupplementaryMaterial.pdf]

RCPedia: A global resource for studying and exploring retrocopies in diverse species

Helena B. Conceição[1,2,#], Rafael L. V. Mercuri[1,2,#] , Matheus P. M. de Castro[1,3], Daniel T. Ohara[1], Gabriela D. A. Guardia[1,*], Pedro A F Galante[1,*]
1 - Hospital Sirio-Libanes, São Paulo, 01308-060, Brazil.
2 - Interunidades em Bioinformática, Universidade de São Paulo, São Paulo 05508-000, Brazil.
3 - Department of Biochemistry, University of São Paulo, São Paulo, Brazil.
# These authors contributed equally.
* Corresponding authors: Gabriela D A Guardia: gguardia@mochsl.org.br and Pedro A F Galante: pgalante@mochsl.org.br

Index

Supplementary Materials and Methods **2**
1. Retrocopies Identification 2
2. Retrocopies Homology 4
3. Retrocopies Expression based on RNA-Seq data 5
Supplementary Figures **5**
Supplementary Figure 1 6
Supplementary Figure 2 7
Supplementary Figure 3 8
Supplementary Figure 4 9
Supplementary Figure 5 11
Supplementary Figure 6 13
Supplementary Figure 7 15
Supplementary Figure 8 16
Supplementary Figure 9 17
References 19

Supplementary Materials and Methods

1. Retrocopies Identification

In this updated version of RCPedia, we have made significant improvements to the retrocopy search process. Firstly, mRNA sequences from coding genes (annotated with XM_ OR NM_ tags) are extracted at specific genomic positions provided by RefSeq using the gffread algorithm(Pertea and Pertea). This helps to address occasional discrepancies between mRNA sequences and genomic annotation highlighted by RefSeqSecondly, we have replaced the BLAT aligner with the LAST aligner(Kiełbasa *et al.*, 2011)lastal -D1000.

Additionally, the pipeline incorporates a series of filters implemented through Python, Perl, and shell scripts, alongside bioinformatics tools like bedtools(Quinlan and Hall, 2010). These filters refine retrocopy identification by considering criteria such as match length, exon-exon boundary determination, alignment position, and exclusion of repetitive elements, resulting in a more accurate retrocopy identification.

Specifically, the filtering process involves:

i) Selecting only alignments from the LAST output that exceed a match length of120 base pairs;

ii) Filtering alignments with a distance greater than 200,000 base pairs from the origin gene of the messenger RNA to eliminate potential chromosomal duplications;

iii) Determining the exon-exon boundary position within the messenger RNA sequence of coding genes and checking for the presence of the last, penultimate, or

antepenultimate exon-exon boundary in the alignment, as the reverse transcriptase processes messenger RNA from the poly-A tail;

iv) Excluding alignments predominantly composed of repetitive elements (greater than or equal to 40%) based on RepeatMasker(RepeatMasker Home Page) annotation, supplemented bysimpleRepeats and windowMasker for non-mammals.

Furthermore, in case of multiple alignments from different messenger RNAs of the same gene, the algorithm selects the alignment with the highest match. In the event of a tie, the one with the highest score (match/(match+mismatch)) is chosen. The algorithm also gathers continuous alignments originating from the same messenger RNA, allowing for the presence of repetitive elements between shorter alignments that are up to 6,000 base pairs apart in the genome.

To mitigate challenges in non-mammalian species, the algorithm incorporates several tailored strategies. Firstly, a distance filter is implemented, restricting retrocopy insertions from the same parental gene to occur no closer than every 500,000 base pairs. This measure reduces false positives without resorting to pre-defined blacklists. Additionally, we address issues stemming from large gene families by implementing two key measures. Retrocopies that overlap with three or more exons of annotated coding genes are removed from consideration. Parental genes harboring more than five retrocopies that overlap with annotated genes from the same gene family. These steps help to streamline the identification process and improve the reliability of results.

In instances where multiple candidates arise from distinct parental genes, the selection is based on the best score, with ties broken randomly.

Lastly, manual analysis is conducted to preserve occurrences of retrocopy insertions within older retrocopies. This meticulous approach adds an extra layer of validation, enhancing the precision and efficiency of retrocopy identification, especially in non-mammalian species with distinct genomic characteristics.

2. Retrocopies Homology

To identify potential orthologous retrocopies, our approach involved retrieving the genomic sequence surrounding each retrocopy (3,000 base pairs upstream and downstream). Pairwise alignments were conducted using the Lastz aligner(Improved pairwise alignment of genomic DNA, 2007), between each retrocopy surrounding region and all other retrocopies and their corresponding regions (3,000 base pairs upstream and downstream) from other species. Filtering criteria were established to ensure robustness. For comparisons between primates, alignment coverage exceeding 60% and identity surpassing 70% were required. For comparisons between non-primates or primates and non-primates species, a more permissive threshold was applied. with coverage above 50% and identity above 60%, acknowledging the anticipated reduced conservation. Additionally, we verified if at least 60% of the retrocopy aligned with the "target" region of the other species in primate comparisons, or at least 50% in non-primate cases. In instances of multiple potential orthologs, preference was given to the alignment with the highest coverage and identity for each retrocopy. Unfortunately, orthologs could not be identified for reptiles, amphibians, fish, and invertebrates (five species) due to the insufficient identity and coverage of the alignments. This highlights the challenges posed by evolutionary divergence in these species groups.

3. Retrocopies Expression based on RNA-Seq data

Retrocopies originating from both humans and mice were linked to corresponding GENCODE IDs. This association facilitated the utilization of data from the GTEx(Consortium *et al.*, 2015) and ARCHS4(Lachmann *et al.*, 2018) consortia to assess retrocopy expression levels. For other species, retrocopy expression quantification was achieved through the application of the Kallisto pseudo-alignment algorithm(Bray *et al.*, 2016) in RNASeq experiments involving diverse tissues. Selected datasets from the NCBI SRA database were utilized for this purpose. The resulting expression estimates underwent normalization, factoring in both the total number of mapped reads and transcript length. This normalization process yielded TPM (transcripts per million mapped reads) values, providing a standardized metric for retrocopy expression across various tissues.

Supplementary Figures

Supplementary Figure 1

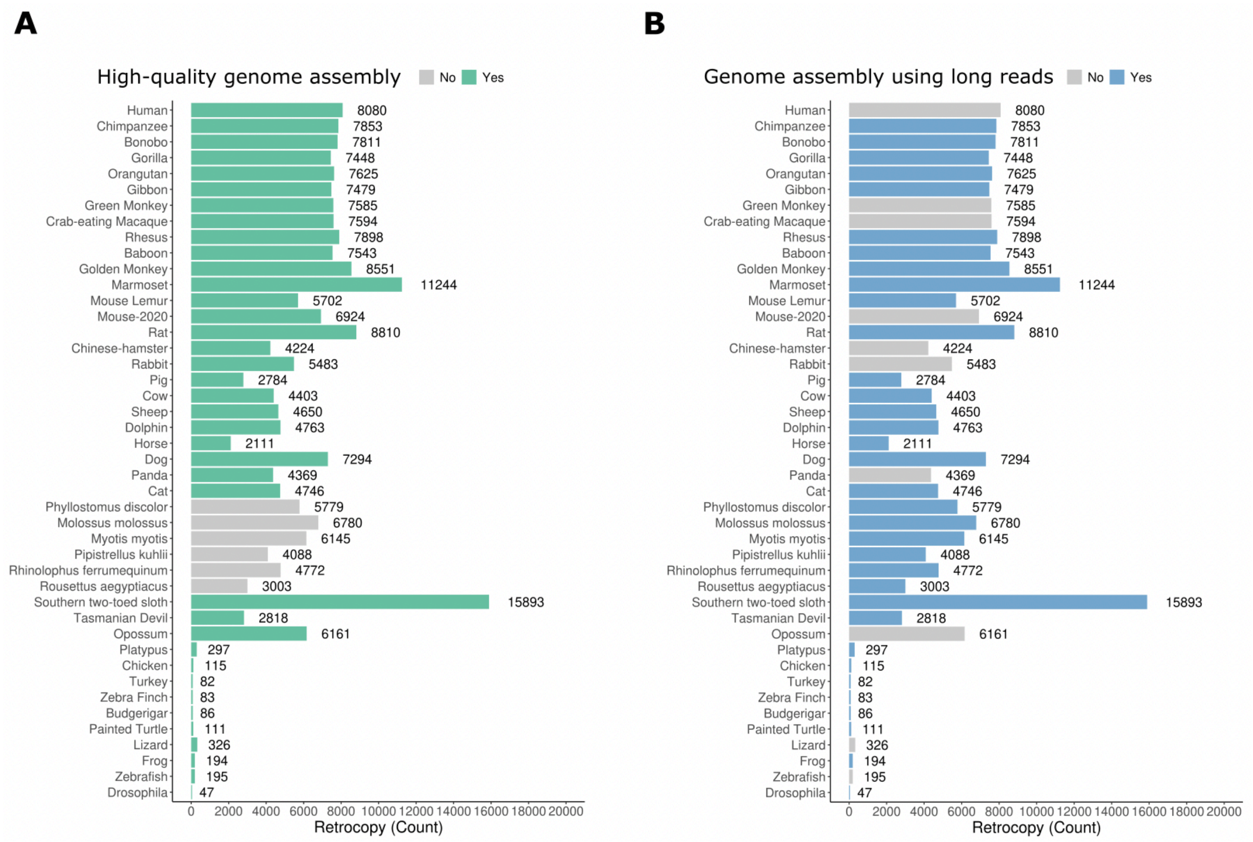

Supplementary Figure 1. Number of retrocopies per species in relation to genome assembly quality and long reads sequencing platforms. A) Species with high-quality genome assemblies are indicated in green, while those without high-quality assemblies are shown in gray. B) Number of retrocopies per species with long-read sequencing used in their genome assemblies are indicated in blue, while those without long-read sequencing are shown in gray.

Supplementary Figure 2

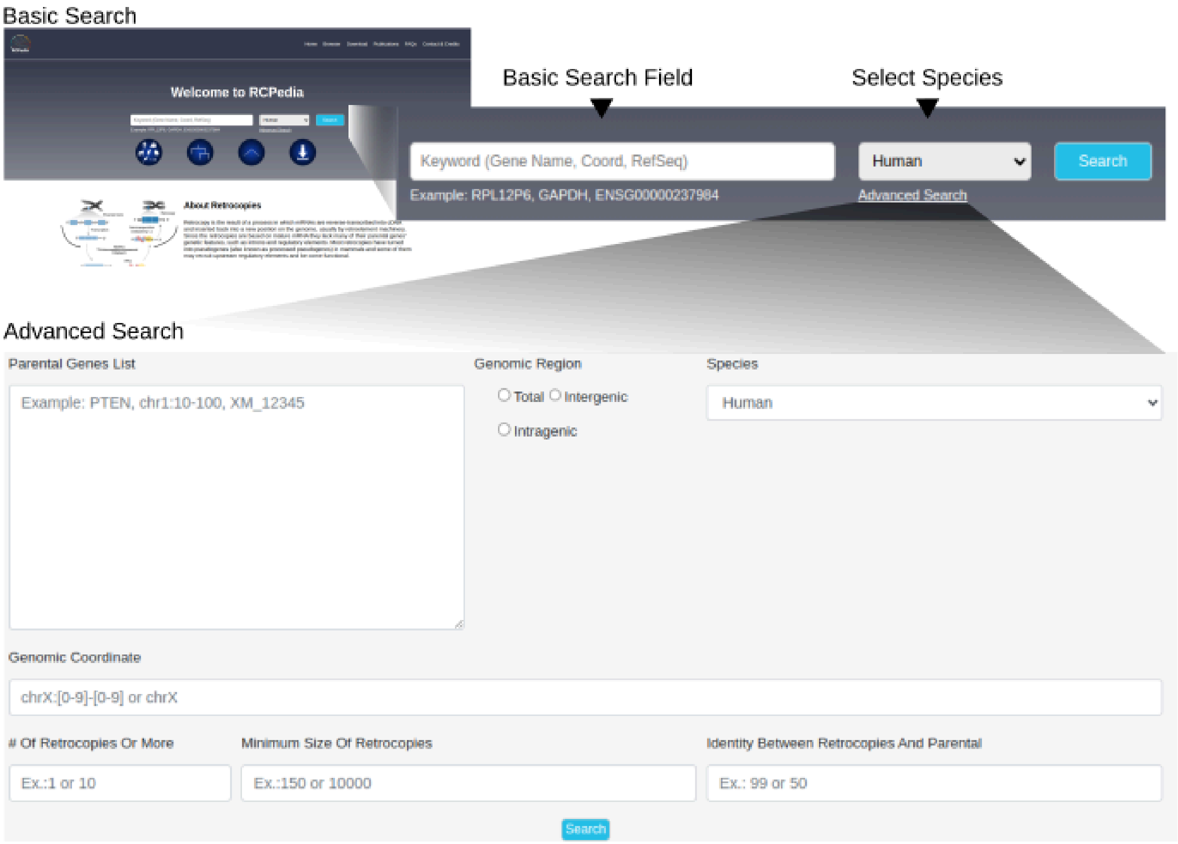

Supplementary Figure 2. RCPedia features both a basic and an advanced query system, designed for ease of use and speed. The basic search functionality allows users to input gene name, retrocopy name, gene Ensembl ID, or transcript RefSeq ID and select the desired species name. Advanced search supports the search of gene name, gene Ensembl ID, or transcript RefSeq ID list within the chosen species. Users can also specify if retrocopies should be intergenic, intragenic, or both. Results can also be narrowed down by genomic coordinates, the number of retrocopies of the parental gene, length of RTC, and RTC-parental identity.

Supplementary Figure 3

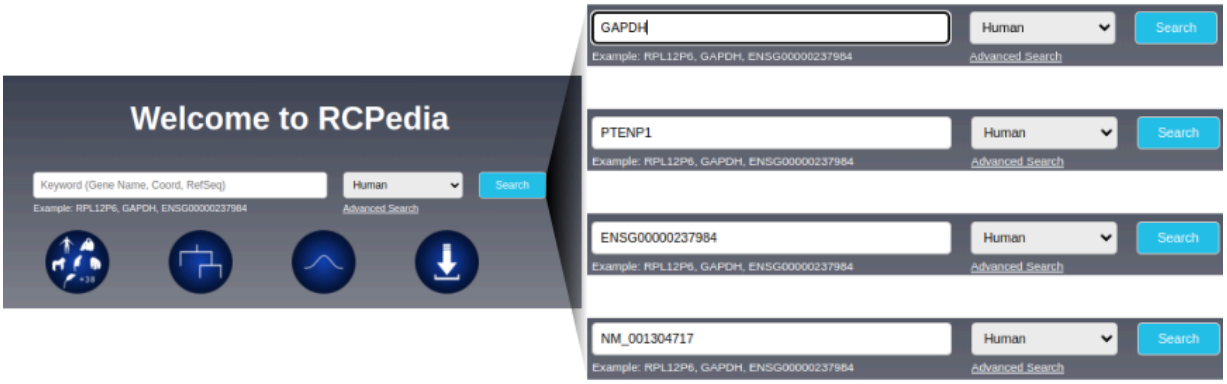

Supplementary Figure 3. Users can efficiently perform searches of retrocopies. Users can query using official gene names, RCPedia given retrocopy names, gene ENSEMBL ID and transcripts RefSeq ID.

Supplementary Figure 4

Summary

Retrocopy Name	PTENP1
Species	*Homo sapiens*
Coordinates (hg38)	chr9:33674359-33677332 UCSC
Coordinates (T2T)	chr9:33692870-33695843 UCSC
Coordinates (hg19)	chr9:33674357-33677330 UCSC
Strand	-
Parental Sequence	NM_001304717.5_2
Parental seq. overlap	2565 bp
Parental seq. overlap (%)	29.5%
Genomic Region	Intergenic
Retrocopy Summary	PTENP1, located on chr9:33674359-33677332, is a retrocopy of the parental gene PTEN. Retrocopies of protein-coding genes, also known as processed pseudogenes, are intriguing genomic elements with implications in genome evolution and diseases. While some retrocopies are non-functional, there are examples of retrocopies (retrogenes) acquiring regulatory roles or exhibiting neofunctionalization unrelated to their parental genes.

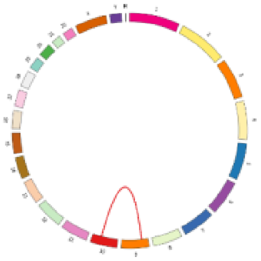

Plot displaying the genomic locations of a **retrocopy (in chr9)** and its respective **parental gene (in chr10)**. Each line represents a retrocopy.

Homology

	Species	Scientific Name	Retrocopy
	Chimpanzee	*Pan troglodytes*	PTENP1
	Gorilla	*Gorilla gorilla*	Without Homology

Expression

The Genotype-Tissue Expression project - (GTEx)

log10(TPM+1) <=> TPM

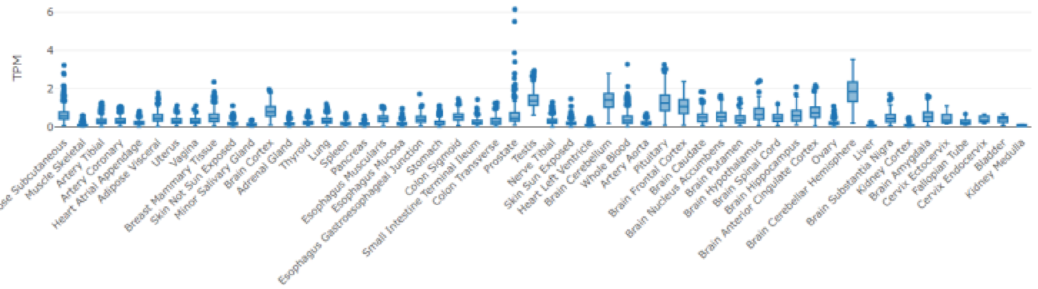

Related Sequence

>PTENP1 Copy FASTA sequence

```
TTTCCATCCTGCAGAGCCCCGCCACCAGCAGCTTCTGCCATCTCTCTCCTCCTTTTTCTTCAGCCACAGGCTCCCAGACAGGACAGCCATCATCAAAGAGATCGTTAGCAGAAACAAAAGGAGATATCAAGAGGATGGATTCGACTTAGACTTGACCTATATTTATCTAAACATTAT
TGCTATGGGATTTCCTGCAGAAAGACTTGAAGGCGTATACAGGAACAATATTGATGATGTAGTAAGGTTTTTGGATTCAAAGCATAAAAACCATTACAAGATACACAATCTTTGTGCTGAAAGACATTATGACACCGCCAAATCTAATTACAGAGTTGCGCAATATCCTTTTTGAAGA
CCATAACCCACCACAGCTAGAACTTATCAAACCCCTTTTGTGAAGATCTTGACCAATGGCTAAGTGAAGATGACAATCATGTTGCAGCAATTCACTGTAAAGCTGGAAAGGGACGAACTGGTATAATGATTTATGCATATTTATTACATCGGGGCAAATTTTTAAAGGCACAAGAGGC
CCTAGATTTCTATGGGGAAGTAAGGACCAGAGACAAAAAGGGAGTAACTATTCCCAGTCAGAGGCGCTATGTGTATTACTATAGCTACCTGGTAAAGAATCATGTGGATTATAGACCAGTGGCACTGTTGTTTCACAAGATGATGTTTGAAACTATTCCAATGTTCAGTGGCGGAAC
TTGCAATCCTCAGTTTGTGGTCTGCCAGCTAAAGGTGAAGATGTATTCCTCCAATTCAGGACCCACACGATGGGAGGACAAGTTCATGTATTTTGAGTTCCCTCAGCCGTTACCTGTGTGTGGTGATATCAAAGTAGAGTTCTTCCACAAACAGAACAAGATGCTAAAAAAGGACAA
AATGTTTCACTTTTGGGTAAATACATTCTTCATACCAGGACCAGAGGAAACCTCAGAAAAAGTAGAAAATGGAAGTCTATGTGATCAAGAAATTGATAGCATTTGCAGTATAGAGCGTGCAGATAATGACAAGGAGTATCTAGTACTTACTTTAACAAAAAATGATCTTGACAAAGC
AAATAAAGACAAAGCCAACCGATACTTTTTCTCCAAATTTTAAGGTGAAGCTGTACTTCACAAAAACAGTAGAGGAGCCGTCAAATCCAGAGGCTAGCAGTTCAACTTCTGTAACACCAGATGTTAGTGACAATGAACCTGATCATTATAGATATTCTGACACCACTGACTCTGATCC
AGAGAATGAACCTTTTGATGAAGATCAGCATACACAAATTACAAAAGTCTGAATTTTTTTTTATCAAGAGGGATAAAACACCATGAAAACAAACTTGAATAAACTGAAAAGGGACCTTTTTTTTTTAATGGCAATAGGACATTGTGTCAGATTACCAGTTATAGAAACAATTCTCTTT
TCGTGACCAATCTTGTTTTACCCTATACATCCACAGGGTTTTGACACTTGTTGTCCAGTTGAAAAAAGGTTGTGTAGCTGTGTCATGTATATACCTTTTTGTGTCAAAAGGACATTTAAAATTCAATTAGGATTAATAAAGATGGCACTTTCCCATTTTATTCCAGTTTTATAAAAA
GTGGAGACAGACTGGTGTGTATACGTAGGAATTTTTTTCCTTTTGTGTTCTGTCACCAACTGAAGTGGCTAAAGAGCTTTGTGATATACTGGTTCACATCATACCCCTTTGCACTTGTGGCAACAGATAAGTTTGCAGTTAGCTAAGAGAAGTTTCTGAAGGGTTTTGCTGCATTCTT
GCATGTATTTGGGTTAGGGGAATGGAGGGAATGCTCAGAAAGGAAATAATTTTATGCTGGACTCTGGACCATATACCATCTCCAGCTATTTACACACACCTTTCTTTAGCATGCTACAGTTATTAATCTGGACATTCGAGGAATTGGCTGCTGTCACTGCTTGTTGTTTTCGCATTT
TTTTAAAAGCATATTGGTGCTAGAAAAGGCAGCTAAAGGAAGTGAATCTGTATTGGGGTACAGGAATGAACCTTCTGCAACATCTTAAGATCCACAAATGAAGGGATATAAAAATAATGTCATAGATAAGAAACACAGCAACAATGACTTAACCATATAAATGTGGAGGCTATCAAC
AAAGAATGGGCTTGAAACATTATAAAAATTGACAATGATTTGTTAAATATGTTTTCTTAATTGTAACGACTTCTCCATCTCCTGTGTAATCAAGGCCAGTGCTGAAAGTCAGATGCTATTAGTACCTACATCAGTCAACAACTTACACTTATTTTACTAGTTTTCAATCATATACCT
GCTGTGAATGCTTCATGTGCTGCCTGCAAGCTTCTTTTTTTCTCATTAAATATAAAATATTTTGTAATGCTAACATGGAGGGACCCGGGCCGGTTTTAAACCTCTCGTCCGCCGCCGCACCCCCCGTGGCCCGGGCTCCGGAGGCCGCTGGAGGAGGCAGCCGTTCGGAGGATTA
TTCGTCTTCTCCCCATTCCGCTGCCGCCGCTGCCAGGCCTCTGGCTGCTGAGGAGAAGCAGGCCCAGTAGCTGCCACCATCCAGCAGCTGCTGCCGCAGCCATTACCCGGCTGCGGTTTTCTCGCTTCTTCTTCGTCTTTTCTAACTGTGCAGCCTCTTCCTCGGCTTCTCCTGAAA
GGGAAGGTGGAAGCCGTGGGCTTGGGCGGGAGCCGGCTGAGGCGCGGCGGCGGCGGCACCTCCCCCTCCTGGAGCGGGGGGAGAAGCGGCGGCGGCGGCGCCCCCGGTGGCCCCGGCGGCTGCAGCTCCAGGGAGGGGGTCTGAGTCGCCTGTCACCATTTCCAGGGCTGGGAACGC
CGGAGAGTTGGTCTCTCCCCTTCTACTGCCTCCAACA
```

Publications

PMID - Link	Title
34799795	Rs7853346 Polymorphism in lncRNA-PTENP1 and rs1799864 Polymorphism in CCR2 are Associated with Radiotherapy-Induced Cognitive Impairment in Subjects with Glioma Via Regulating PTENP1/miR-19b/CCR2 Signaling Pathway.
34165715	Studying the Oncosuppressive Functions of PTENP1 as a ceRNA.

Supplementary Figure 4. Comprehensive data provided by RCPedia regarding retrocopies and parental genes. The retrocopy page showcases details such as the retrocopy name, species, genomic coordinate (with USCS link), insertion strand, originating transcript RefSeq ID, RTC-Parental coverage, genomic context (inter or intragenic), and a brief retrocopy summary. Moreover, the resource offers detailed information on genomic locations in the form of a graphical visualization of RTC and Parental gene coordinates through Circos plot. Additionally, RCPedia provides orthology information with other species, expression data quantified in Transcripts per Million and logarithmic values, fasta sequence, and publications pertaining to each retrocopy.

Supplementary Figure 5

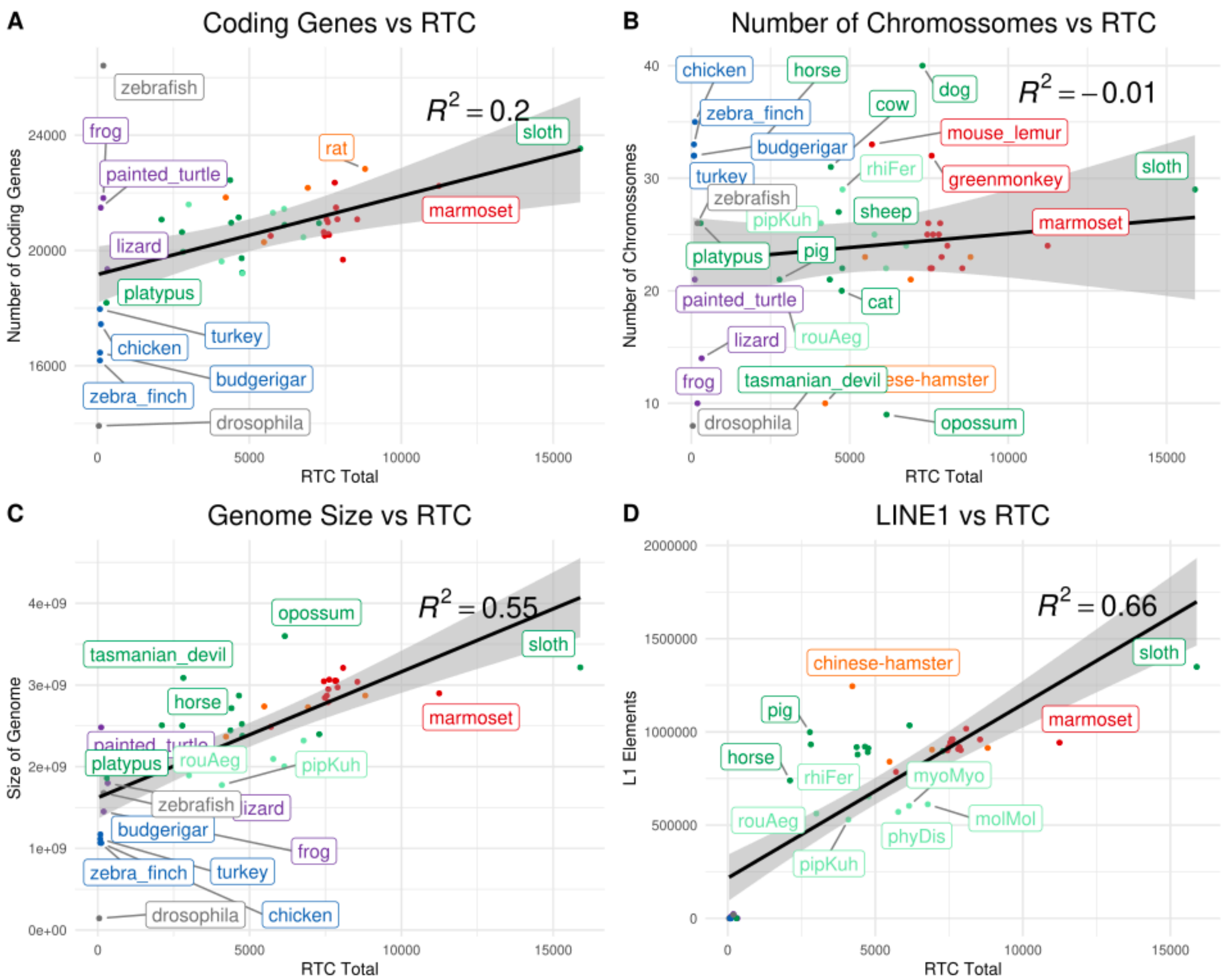

Supplementary Figure 5. Correlation analysis of the number of retrocopies (RTC) with various genomic features. A) Coding Genes vs RTC. The scatter plot shows the relationship between the total number of coding genes and the total number of retrocopies (RTC) for each species. B) Number of chromosomes vs RTC. The scatter plot illustrates the relationship between the number of chromosomes and the total number of retrocopies (RTC) for each species. C) Genome size vs RTC. The scatter plot presents the relationship between the genome size and the total number of

retrocopies for each species. D) LINE1 Elements vs RTC. The scatter plot shows the relationship between the number of LINE1 elements and the total number of retrocopies for each species. The R^2 value of 0.67 indicates a strong positive correlation. Species are labeled and color-coded. The shaded areas in each plot represent the confidence intervals for the regression lines.

Supplementary Figure 6

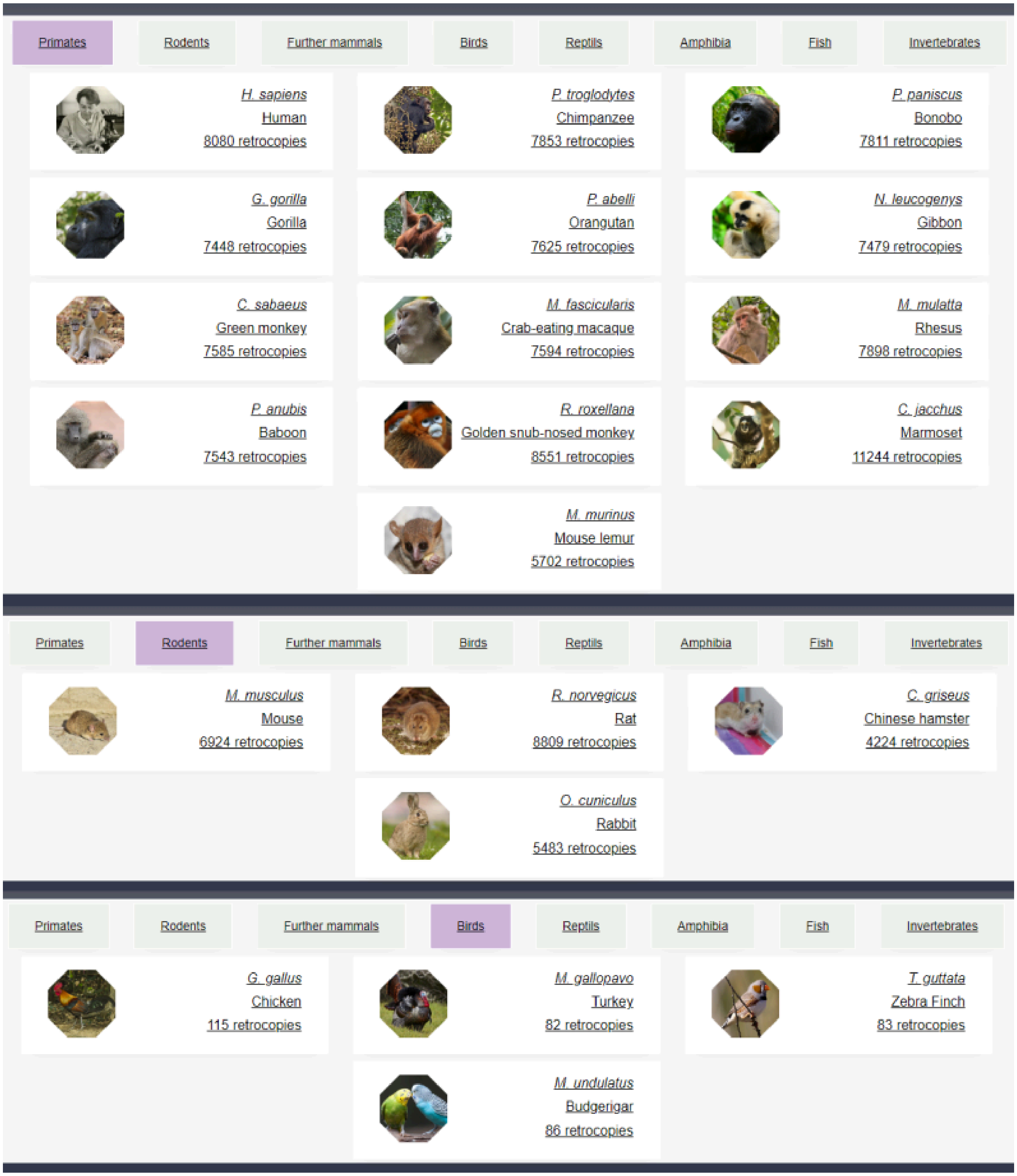

Supplementary Figure 6. The Browser pages grant access to all retrocopies for each species. This feature simplifies retrocopy number searches by species. Species are categorized into eight main groups: Primates, Rodents, Further Mammals, Birds, Reptiles, Amphibia, Fish, and Invertebrates. Essential information such as scientific name, common name, and the total number of retrocopies are readily visible. Clicking on a specific species redirects the users to the corresponding species pages, where all retrocopies are listed.

Supplementary Figure 7

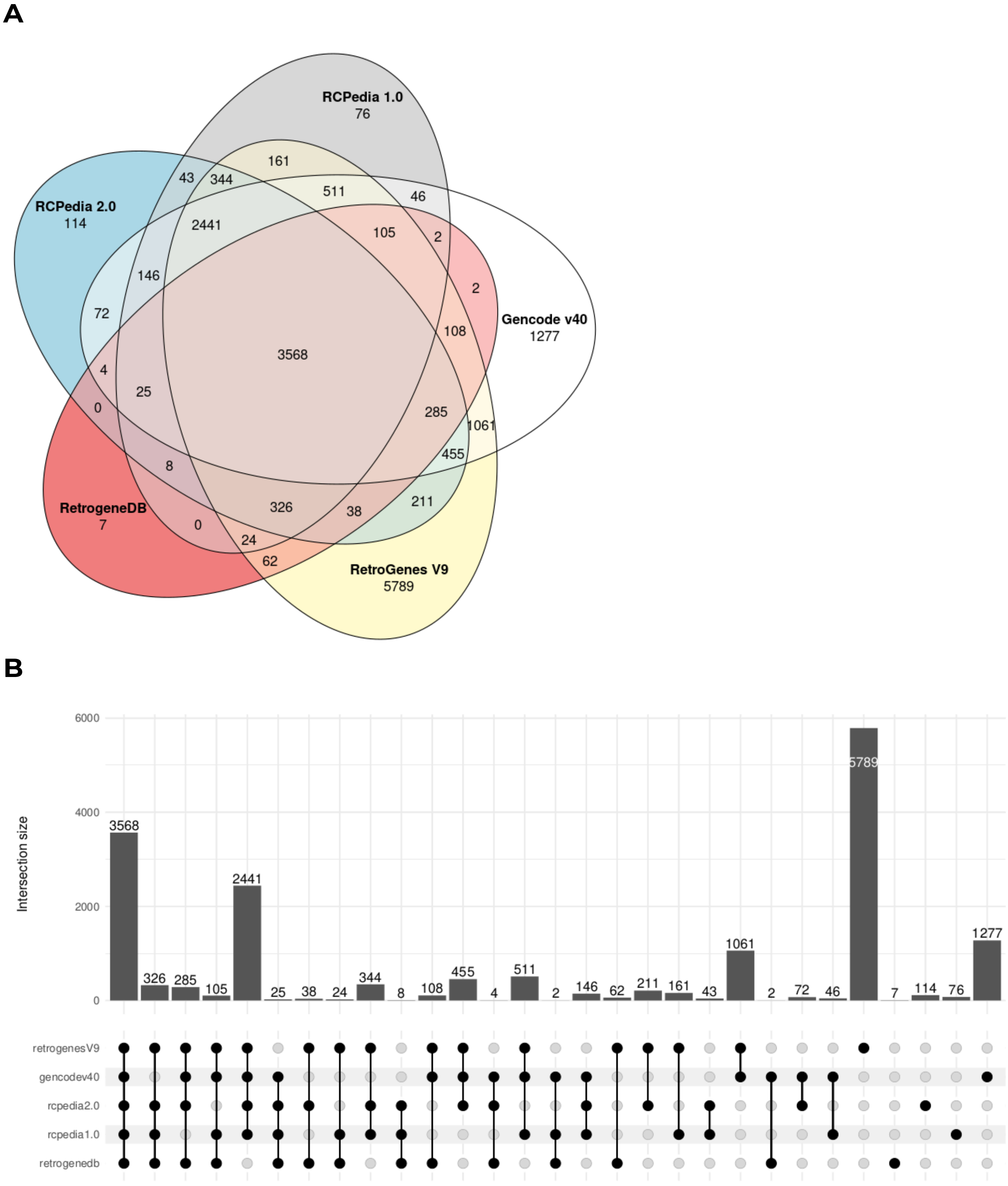

Supplementary Figure 7. A comparative analysis between RCPedia 2.0 and four other retrocopy annotations was conducted, including RCPedia 1.0,

RetrogeneDB, RetroGenes V9, and Gencode V40. A) Venn diagram illustrating the overlap and unique sets of retrocopies identified by RCPedia 2.0 and other databases. (B) Upset plot showing the intersection sizes of retrocopies across the same databases. Each bar represents the number of retrocopies found in a specific combination of databases, providing a clear visualization of the common and unique retrocopies among RCPedia 2.0, RCPedia 1.0, RetrogeneDB, Gencode v40, and RetroGenes V9.

Supplementary Figure 8

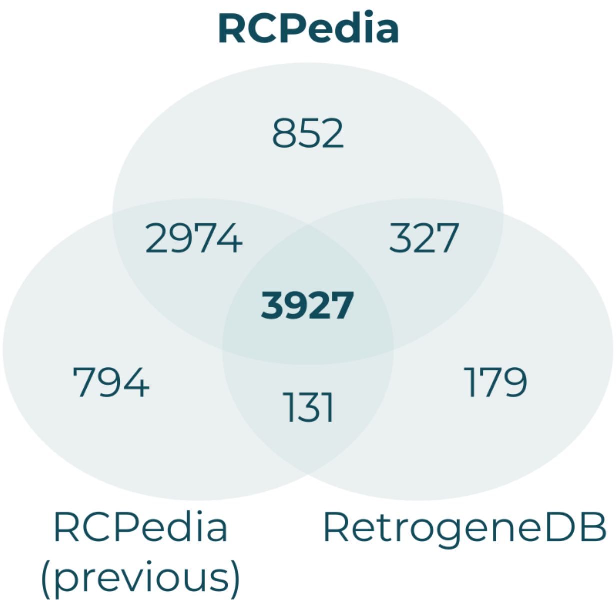

Supplementary Figure 8: Comparison of Retrocopies Identified by RCPedia and RetrogeneDB. The Venn diagram illustrates the overlap and unique sets of retrocopies identified by the new version of RCPedia, the previous version of RCPedia, and RetrogeneDB.

Supplementary Figure 9

A) Parental information

Summary

Gene Name	Gapdh
Specie	*Rattus norvegicus*
Full Name	glyceraldehyde-3-phosphate dehydrogenase
Also known as	BARS-38|Gapd
Coordinate	chr4:157676336-157680322
Strand	-
Gene summary	This gene encodes a member of the glyceraldehyde-3-phosphate dehydrogenase protein family. A similar protein in human and mouse has been identified as a moonlighting protein based on its ability to perform mechanistically distinct functions. The encoded protein was originally identified as a key glycolytic enzyme that converts D-glyceraldehyde 3-phosphate (G3P) into 3-phospho-D-glyceroyl phosphate. Subsequent studies in human and mouse have assigned a variety of additional functions to the protein including nitrosylation of nuclear proteins. Many pseudogenes similar to this locus are found throughout the rat genome. [provided by RefSeq, Jan 2014]

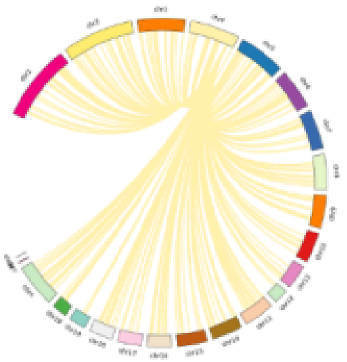

Plot displaying the genomic locations of a retrocopy and its respective parental gene (in chr4). Each line represents a retrocopy.

Retrocopy(s) from Gapdh

Retroname	Coord	Strand	Genomic Region	ENSG	
GapdhP1	chr1:105780971-105782338	-	Intragenic	N/A	UCSC
GapdhP2	chr1:107155915-107156995	-	Intergenic	N/A	UCSC
GapdhP3	chr1:137513948-137515169	-	Intergenic	N/A	UCSC
GapdhP4	chr1:167654597-167655479	+	Intergenic	N/A	UCSC

B) Retrocopy information

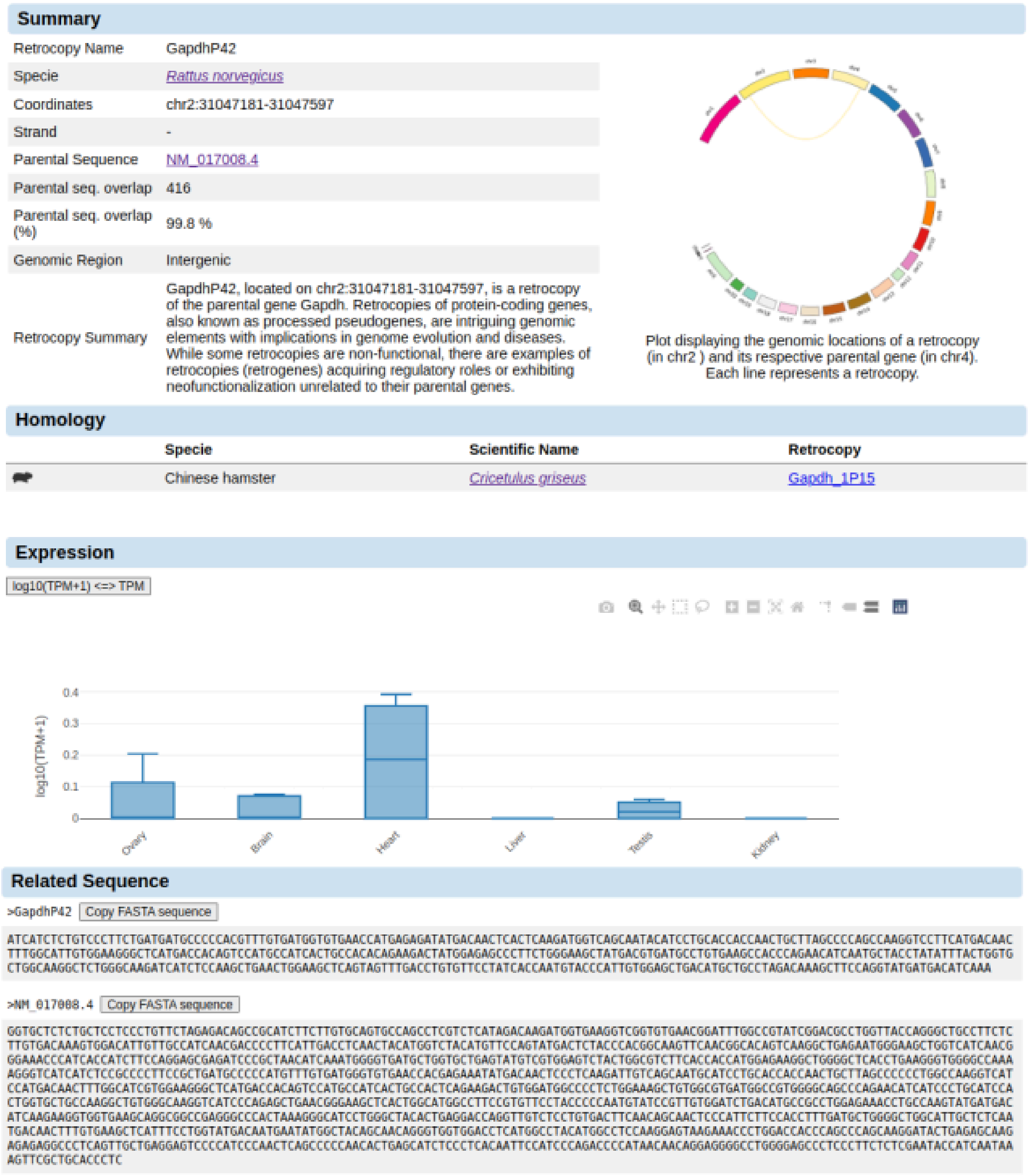

Supplementary Figure 9. Gapdh and its retrocopies in *Rattus norvegicus*. A) The parental gene Gapdh page in RCPedia. The page showcases details such as the parental gene name, species, full gene name, synonyms, genomic coordinate, transcription strand, gene summary from NCBI, and detailed information about

the Parental gene and all of its retrocopies in the Circos plot (right). Below are listed all of Gapdh’s Retrocopies. B) Page of retrocopy GapdhP42, showing: retrocopy name, species, genomic coordinate, insertion strand, originating transcript RefSeq ID, RTC-Parental coverage, genomic context (inter or intragenic), a brief retrocopy summary, a Circos plot, orthology information with other species, expression data (in TPM), and fasta sequence.

References

Bray,N.L. *et al.* (2016) Near-optimal probabilistic RNA-seq quantification. *Nat. Biotechnol.*, **34**, 525–527.

Consortium,T.G. *et al.* (2015) The Genotype-Tissue Expression (GTEx) pilot analysis: Multitissue gene regulation in humans. *Science*, **348**, 648–660.

Improved pairwise alignment of genomic DNA (2007) **3299002**.

Kiełbasa,S.M. *et al.* (2011) Adaptive seeds tame genomic sequence comparison. *Genome Res.*, **21**, 487–493.

Lachmann,A. *et al.* (2018) Massive mining of publicly available RNA-seq data from human and mouse. *Nat. Commun.*, **9**, 1366.

Pertea,G. and Pertea,M. GFF Utilities: GffRead and GffCompare [version 1; peer review: 2.

Quinlan,A.R. and Hall,I.M. (2010) BEDTools: a flexible suite of utilities for comparing genomic features. *Bioinformatics*, **26**, 841–842.

RepeatMasker Home Page
